# Bioinformatics and experimental validation of ferroptosis-related genes in steroid-induced osteonecrosis of the femoral head

**DOI:** 10.3389/fmolb.2025.1578755

**Published:** 2025-05-12

**Authors:** Ming-gang Guo, Chen-fei Yang, Fa Yuan, Tao Yang, Ping-yuan Luo, Yu-bai He, Shuan Yang, Feng Chen, Wei Li, Zhi-wei Feng

**Affiliations:** ^1^ Department of Orthopaedics, Beijing Anzhen Nanchong Hospital, Capital Medical University and Nanchong Central Hospital, Nanchong, China; ^2^ School of Nursing, North Sichuan Medical College, Nanchong, China; ^3^ Department of Orthopaedics, Nanjiang County People’s Hospital, Bazhong, China

**Keywords:** steroid-induced osteonecrosis of the femoral head, ferroptosis, WGCNA, SsGSEA, diagnostic biomarker

## Abstract

**Background:**

Steroid-induced osteonecrosis of the femoral head (SONFH) is a progressive condition that causes increasing disability. It is thought to result from reduced blood flow and oxygen levels in the femoral head, with reactive oxygen species (ROS) playing a key role in triggering ferroptosis. However, the role of ferroptosis in SONFH progression remains underexplored. This study aimed to identify and validate key genes associated with ferroptosis in SONFH using bioinformatics.

**Methods:**

The study analyzed the SONFH dataset GSE123568, which includes data from 30 SONFH patients and 10 controls. Weighted gene co-expression network analysis (WGCNA) was used to identify differentially expressed genes (DEGs) between the SONFH and control groups. Core genes were identified by intersecting DEGs with ferroptosis-related genes retrieved from FerrDb V2. The diagnostic performance of the key genes was assessed using the receiver operating characteristic (ROC) curve, and a predictive nomogram model was developed. Interaction analysis of these genes was conducted to explore their link with immune infiltration. The expression of these genes in bone tissue from SONFH patients was validated. Finally, drug-protein interactions were predicted using the DSigDB database.

**Results:**

Differential expression analysis identified 384 DEGs, which were significantly involved in inflammatory pathways. WGCNA revealed four key genes after intersecting DEGs with relevant module genes and ferroptosis-related genes. A nomogram model based on these genes demonstrated strong reliability and validity. Immune infiltration analysis showed significant differences between SONFH patients and controls, with notable associations between immune cell infiltration and the expression of the four core genes. Validation through quantitative real-time PCR (qRT-PCR) and Western blot confirmed that the expression of GCLC, GABARAPL2, CISD2, and NCOA4 was significantly lower in SONFH bone tissue compared to controls (*P* < 0.05). Additionally, potential therapeutic drugs targeting these genes, including Diethyl sulfate, Meloxicam, and NIMUSTINE, were predicted.

**Conclusion:**

This study identifies GABARAPL2, CISD2, NCOA4, and GCLC as potential diagnostic biomarkers associated with immune cell infiltration in SONFH, offering new insights for future research and clinical applications.

## Introduction

Glucocorticoids have been used to treat numerous connective tissue disorders, including systemic lupus erythematosus, nephrotic syndrome, and post-transplantation complications (Tanaka et al., 2010; Hodson and Alexander, 2008; Tönshoff et al., 2005). One well-known consequence of steroid use is steroid-associated osteonecrosis, or SONFH (Huang et al., 2016). SONFH typically affects young and middle-aged individuals, is often bilateral, and involves severe necrosis, frequently leading to significant disability (Fukushima et al., 2010). Various theories have been proposed regarding the pathogenesis of SONFH, such as osteoporosis, lipid metabolism disorders, vasculopathy, intraosseous hypertension, and glucocorticoid cytotoxicity (Li et al., 2006; Drescher et al., 2011; Takano-Murakami et al., 2009; Siler et al., 2002; Miyanishi et al., 2002; Liu et al., 2005). However, the precise mechanism remains poorly understood.

Growing evidence suggests that steroids trigger apoptosis in osteocytes, osteoblasts, and chondrocytes, providing a cytological basis for SONFH (Zalavras et al., 2003). Additionally, oxidative stress (OS) plays a key role in SONFH pathogenesis. Steroids increase ROS levels, worsening oxidative stress (Ichiseki et al., 2005). Mitochondrial dysfunction is central to this process, releasing excess ROS and apoptosis-inducing factors. These events accelerate telomere shortening, disrupt cell cycle progression, and activate critical signaling pathways, including p53 and p38MAPK, ultimately causing apoptosis and cellular senescence (Sung et al., 2018; Yu et al., 2013; Wei et al., 2010). Ferroptosis, a newly identified form of cell death, differs from apoptosis, necrosis, and autophagy. It involves iron-dependent lipid peroxidation and ROS buildup (Fang et al., 2021; Zheng et al., 2023; Ye et al., 2021; Xie et al., 2016). Excess iron can induce ferroptosis through ROS generated by the Fenton reaction (Yang et al., 2014; Xie et al., 2016; Tang et al., 2021). In this reaction, Fe^2+^ reacts with H_2_O_2_ from mitochondrial respiration, producing hydroxyl radicals (•OH) that damage DNA, proteins, and lipids (Kajarabille and Latunde-Dada, 2019; Healy et al., 2021). Additionally, reduced glutathione (GSH) levels or GPX4 inactivation can promote ferroptosis by increasing lipid peroxidation and ROS accumulation (Yang et al., 2014; Xie et al., 2016; Tang et al., 2021). Ferroptosis-related genes inhibit osteogenic differentiation of bone marrow mesenchymal stem cells (BMSCs) (Lin et al., 2022), contributing to osteoporosis (He et al., 2022). Our previous studies found that TIMP1 affects high glucose and high-fat (HGHF)-induced osteoblast ferroptosis by regulating the stability of TFRC protein, thereby influencing the development of osteoporosis (Peng et al., 2024). Other studies indicate that high-dose glucocorticoids reduce BMSC proliferation and osteogenesis, increasing apoptosis and worsening femoral head necrosis (Wang et al., 2022; Tan et al., 2012; Shen et al., 2018). These findings highlight ferroptosis as a potential therapeutic target for SONFH, although its exact mechanisms in SONFH are not yet fully understood.

In this study, we combined SONFH-related DEGs from the GEO database with ferroptosis-related genes from the FerrDb database and conducted a bioinformatics analysis. We identified ferroptosis-related DEGs and used them to build a diagnostic model (nomogram) for SONFH. We then validated and explored potential biomarkers through *in vitro* experiments. This study’s innovation is the identification of biomarkers linked to SONFH, filling a gap in the literature on the molecular mechanisms of ferroptosis in SONFH and offering new therapeutic targets for the disease.

## Materials and methods

### Data processing

The SONFH dataset, GSE123568, was downloaded from the NCBI Gene Expression Omnibus (GEO) database (https://www.ncbi.nlm.nih.gov/geo/), and includes data from 30 SONFH patients and 10 non-SONFH controls. The dataset consists of normalized gene expression data generated using the Affymetrix Human Genome U133 Plus 2.0 Array platform (accession number: GPL570). Preprocessing and normalization of raw data were performed using the R package affy, which included background correction, quantile normalization, and summarization of probe-level data to gene-level expression values. The ferroptosis gene set was obtained from FerrDb V2 (https://www.zhounan.org/ferrdb/), a widely used resource in ferroptosis-related analyses. DEGs were identified using the Limma package (Ritchie et al., 2015), with the following cutoff criteria: |logFC| > 1 and adjusted *P* value <0.05. The |logFC| > 1 cutoff was selected to identify genes with biologically significant expression changes, as this threshold is commonly used to capture meaningful alterations in gene activity. An adjusted *P* value <0.05 was applied to control for the false discovery rate (FDR) and ensure the statistical significance of the DEGs. Expression heatmaps were generated using the R package heatmap.

### Functional enrichment analysis of DEGs

Gene Ontology (GO) analysis (Gene Ontology Consortium, 2015) was used to describe gene and protein function, molecular function (MF), including biological process (BP), and cellular component (CC). The Kyoto Encyclopedia of Genes and Genomes (KEGG) is a comprehensive database that integrates genomics, biological processes, and disease information (Kanehisa and Goto, 2000). To explore the functions and pathways of DEGs, we used the “clusterProfiler” package (Yu et al., 2012) to perform GO and KEGG analyses with significance criteria (*P* < 0.05). Multiple testing corrections were performed using the False Discovery Rate (FDR) method to control for false positives. Using the “clusterProfiler” R package, Gene Set Enrichment Analysis (GSEA) (Subramanian et al., 2005) was performed with the immunologic signature gene set (C7 gene sets) from the Molecular Signatures Database (MSigDB, https://www.gsea-msigdb.org/), with FDR correction applied.

### Collection of ferroptosis-related genes

Ferroptosis-related genes, including suppressor, driver, and marker genes, were sourced from the FerrDb database (http://zhounan.org/ferrdb/current/) (Zhou and Bao, 2020). The junction of the ferroptosis-related genes and the differentially expressed genes was shown using Venn diagrams.

### Construction of WGCNA

The WGCNA method (Langfelder and Horvath, 2008) facilitates the comprehensive investigation of gene set expression. Utilizing the “WGCNA” R package, gene networks were developed and modularized at various stages. Initial clustering of samples was performed to detect significant outliers. Then, automated procedures created co-expression networks. Modules were identified through hierarchical clustering combined with dynamic tree cutting. To correlate modules with clinical traits, module membership (MM) and gene significance (GS) were calculated. Hub modules were characterized by the highest Pearson correlation of MM and a *P*-value ≤0.05. High connectivity within modules and their clinical relevance were indicated by MM values >0.8 and GS values >0.2, respectively. Information from these modules was further analyzed for subsequent research.

### Construction of PPI network

GeneMANIA (Franz et al., 2018) (http://www.genemania.org) enables the creation of Protein–Protein Interaction (PPI) networks, facilitating the prediction of gene functions and identification of genes with similar effects. Numerous bioinformatics techniques, including as physical interactions, co-expression, co-localization, gene enrichment analysis, genetic relationships, and site predictions, are employed by this network integration program. The PPI networks of signature genes were examined in this study using GeneMANIA.

### Immune infiltration analysis using ssGSEA

Single-sample gene set enrichment analysis (ssGSEA) (Subramanian et al., 2005) was used to quantify the infiltration scores of 16 immune cell types and the activities of 13 immune-related pathways. The gene sets for immune-related pathways and immune cell types were obtained from the C7 immunologic signature gene sets in the Molecular Signatures Database (MSigDB) (https://www.gsea-msigdb.org/gsea/msigdb/index.jsp). The infiltration score represents the relative abundance of each immune cell type or pathway in a given sample. The Spearman rank correlation coefficient was computed using the “corrplot” software to investigate the association between immunological state and certain genes.

### Clinical specimens

Nine femoral head tissue samples were collected from SONFH patients at Nanchong Central Hospital. All patients met the clinical and imaging diagnostic criteria for SONFH, including specific radiological features and clinical history. The inclusion criteria for the SONFH group were: (1) a confirmed diagnosis of SONFH based on clinical and imaging evaluations, (2) no history of other major systemic diseases, and (3) history of corticosteroid use. Exclusion criteria included: (1) patients with secondary osteonecrosis due to trauma, infection, or other diseases, (2) patients who had received hip surgery before the tissue collection, and (3) those with any malignancies. Additionally, nine femoral head tissue samples from patients undergoing total hip replacement surgery, with no history of SONFH, were obtained as the control group (CON). The control group inclusion criteria were: (1) patients undergoing hip replacement for primary osteoarthritis, (2) no clinical or radiological signs of SONFH, and (3) no history of corticosteroid use. Sample IDs and demographic details for both groups are outlined in Supplementary Table S1.

### Hematoxylin-eosin (H&E) staining

Bone tissue samples were collected and fixed in 10% neutral buffered formalin for 48 h. The fixed tissues were embedded in paraffin and sectioned. Sections were deparaffinized in xylene, gradually dehydrated in ethanol, and stained with hematoxylin and eosin. After differentiation, sections were counterstained with eosin. The stained sections were then dehydrated in graded ethanol and mounted with neutral resin. Finally, the specimens were examined under a fluorescence microscope (Olympus, Japan).

### RNA extraction and qRT-PCR

Using the Trizol reagent (Invitrogen, Carlsbad, CA, United States), total RNA was isolated from bone tissue (*n* = 9). The PrimeScript RT Reagent Kit and gDNA Eraser (Takara Bio, Inc., Kyoto, Japan) were used to reverse transcribe the extracted RNA into complementary DNA (cDNA). On a CFX96 machine (Bole, Inc., CA, United States), qRT-PCR was carried out using the SYBR Green qPCR Mix (Takara Bio, Inc., Japan). The 2^−ΔΔCq^ technique was used to quantify the target gene expression levels normalized to GAPDH (Livak and Schmittgen, 2001). GAPDH was selected as the reference gene after assessing the expression stability of other commonly used housekeeping genes, such as ACTB and 18S, in the dataset. GAPDH demonstrated the most stable expression under the experimental conditions, making it the most suitable normalization gene for this study. Table 1 lists the primer sequences for qRT-PCR.

**TABLE 1:** The sequence of primers.

Gene	Forward and reverse primer
GABARAPL2	F: 5′ ACTCGCTGGAACACAGATGC 3′
	R: 5′ TCTGAGAGCCTGAGACCTTTT 3′
CISD2	F: 5′ GTGGCCCGTATCGTGAAGG 3′
	R: 5′ CTAGCGAACCCGGTAATGCTT 3′
NCOA4	F: 5′ CAGCAGCTCTACTCGTTATTGG 3′
	R: 5′ TCTCCAGGCACACAGAGACT 3′
GCLC	F: 5′ GGGGTGACGAGGTGGAGTA 3′
	R: 5′ GTTGGGGTTTGTCCTCTCCC 3′
GAPDH	F: 5′ TGTGTCCGTCGTGGATCTGA 3′
	R: 5′ TTGCTGTTGAAGTCGCAGGAG 3′

### Protein extraction and Western blot analysis

RIPA buffer (Beyotime, China) was used to extract proteins from bone tissue (*n* = 3), and Beyotime’s BCA Protein Assay Kits were used to measure the proteins. After that, the proteins were heated for 10 min and mixed in a 1:3 ratio with loading buffer to denature them. The proteins were separated using SDS-PAGE and then put onto PVDF membranes (Millipore, United States). After blocking PVDF membranes with 5% BSA for an hour at 4°C, the primary antibodies against GCLC (Abcam, ab207777), GABARAPL2 (Abcam, ab126607), CISD2 (Proteintech, 13318-1-AP), NCOA4 (Abcam, ab314553), and β-actin (Proteintech, 20536-1-AP) were added, and the membranes were incubated for an entire night at 4°C. Horseradish peroxidase-linked secondary antibody (Proteintech, SA00001-2) was applied to the membranes for an hour at room temperature following the incubation of the primary antibody. β-actin was used as the internal control for Western blot normalization to ensure equal protein loading. An ECL kit (Biosharp, BL520A, China) was used to observe the signal bands, and ImageJ software (Bethesda, MD, United States) was used to analyze the signal bands for gray values.

### Evaluation of applicant drugs

Networks of protein-drug interactions have grown in significance during the drug development process (Yan et al., 2018). In this study, we identified 21 potential drug candidates from transcriptomic signatures in the DSigDB database (Yoo et al., 2015) using the Enrichr tool (Kuleshov et al., 2016). Based on their adjusted *P*-values, the top 10 medications were selected. Because they could have therapeutic potential for treating SONFH, suggested as potential modulators of the hub genes. The effective medications for the hub genes found in the DSigDB database are shown in Table 2.

**TABLE 2:** List of the suggested drugs for SONFH.

Name	Adjusted *P*-value	Chemical formula	Genes	Functional annotation
Diethyl sulfate CTD 00000613	0.0372	C_4_H_10_O_4_S	GCLC	Induce apoptosis
Meloxicam CTD 00002644	0.0372	C_14_H_13_N_3_O_4_S_2_	GCLC	Anti-inflammatory
IMD-0354 CTD 00004363	0.0372	C_15_H_8_C_l_F_6_NO_2_	GCLC	Anti-inflammatory
Penconazole CTD 00003093	0.0372	C_13_H_15_C_l2_N_3_	NCOA4	Antifungal
NIMUSTINE CTD 00007067	0.0372	C_9_H_13_C_l_N_6_O_2_	GCLC	Inhibit DNA replication
Isorhamnetin CTD 00002092	0.0372	C_16_H_12_O_7_	GCLC	Anti-oxidant
Cyclosporin A CTD 00007121	0.0372	C_62_H_111_N_11_O_12_	GABARAPL2/GCLC/NCOA4/CISD2	Anti-inflammatory
Rosuvastatin CTD 00003899	0.0372	C_22_H_28_FN_3_O_6_S	GCLC	Reduce cholesterol synthesis
VERLUKAST CTD 00002469	0.0372	C_26_H_27_C_l_N_2_O_3_S_2_	GCLC	Anti-inflammatory
NICKEL SULFATE CTD 00001417	0.0372	NiO_4_S	GCLC/NCOA4	Induce oxidative stress

### Statistical analysis

Prism 8.0 was used to perform statistical analysis (GraphPad Software, Inc., San Diego, CA, United States). For normally distributed data, *t*-tests were used to compare two groups, and for multigroup comparisons, one-way ANOVA was used after testing for variance homogeneity using Levene’s test. Shapiro-Wilk tests were applied to assess the normality of the data. *P* < 0.05 was considered the threshold for statistical significance, and the results are presented as mean ± standard deviation. To ensure robustness and repeatability, each experiment was conducted in triplicate.

## Results

### DEGs screening and data preprocessing

**FIGURE 1:**
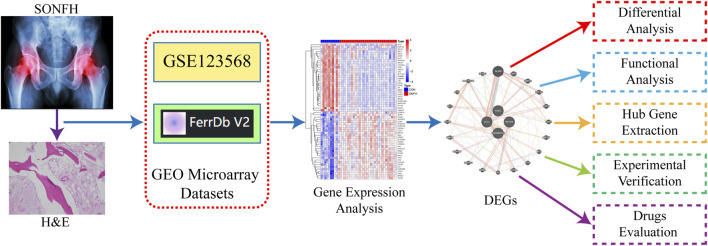
Diagrammatic representation of the study’s general procedure.

**FIGURE 2:**
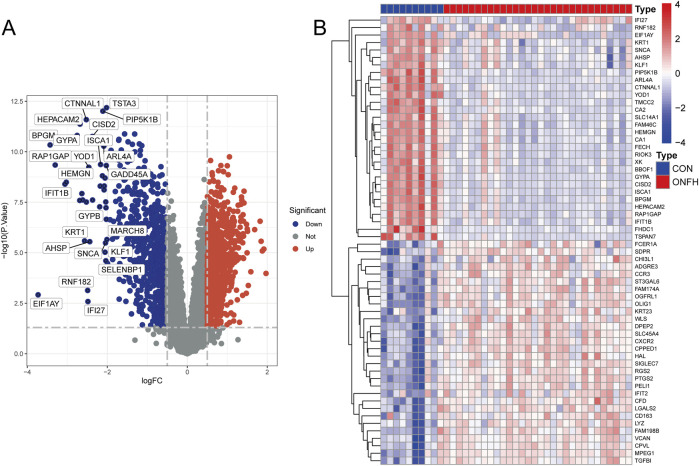
Preparation of the DEGs’ data. **(A)** Volcano plot showing the distribution of differentially expressed genes (DEGs). The color coding in the volcano plot represents the statistical significance, where red indicates highly significant DEGs, and blue indicates less significant DEGs. **(B)** Heatmap illustrating the expression patterns of the top 50 DEGs. The colors represent the relative expression levels of each gene across the samples: red indicates higher expression, while purple indicates lower expression. The columns represent different samples, and the rows represent genes. Log_2_-transformed expression values are presented in the heatmap.

Figure 1 shows the workflow for the research. 384 genes were found to be DEGs using the criterion of an adjusted *P*-value <0.05 and |logFC| > 1, with 265 genes downregulated and 119 genes upregulated. Figure 2A shows the volcano plots of DEGs, and Figure 2B shows a heat map of the top 50 genes.

### Function enrichment analysis

**FIGURE 3:**
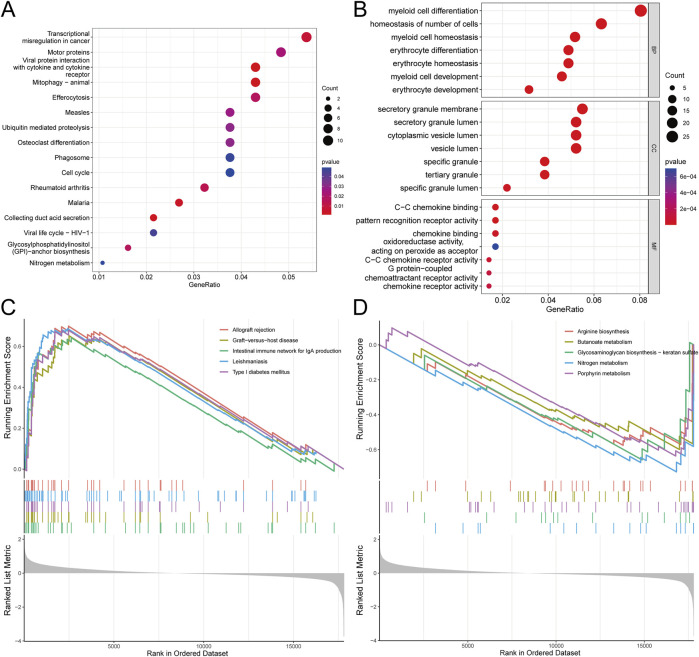
Functional analysis of key module genes integrated with DEGs. **(A)** KEGG pathway analysis showing the significant pathways enriched by the key module genes and DEGs. The color coding in the pathway diagram indicates the degree of enrichment, where red represents pathways with higher significance (*p*-value <0.05), and blue indicates lower significance (*p*-value >0.05). **(B)** GO analysis illustrating the enriched biological processes (BP), molecular functions (MF), and cellular components (CC) for the key module genes and DEGs. The sizes of the circles represent the number of genes involved, and the color gradient reflects the significance of enrichment, with darker colors indicating more significant terms (*p*-value <0.05). **(C,D)** GESA analysis. For GESA, the statistical thresholds applied are *p*-value <0.05, FDR <0.25, and NES >1.5.

Understanding the signaling pathways, biological processes, and interrelationships involved in DEGs is crucial for elucidating the pathogenesis of SONFH. KEGG pathway analysis revealed that these DEGs were primarily associated with mitophagy, viral protein interactions with cytokines and cytokine receptors, transcriptional misregulation in cancer, and osteoclast differentiation (Figure 3A). GO enrichment analysis indicated that DEGs were significantly enriched in biological processes (BP) related to myeloid cell development and reactive oxygen species metabolism, cellular components (CC) such as the secretory granule membrane and specific granules, and molecular functions (MF) including C-C chemokine binding and pattern recognition receptor activity (Figure 3B). Additionally, GSEA suggested that SONFH development may be linked to allograft rejection, arginine biosynthesis, the NOD-like receptor signaling pathway, the chemokine signaling pathway, Th1 and Th2 cell differentiation, and the NF-kappa B signaling pathway (Figures 3C,D). The complete GSEA analysis results are provided in Supplementary Table S2.

### WGCNA analysis

**FIGURE 4:**
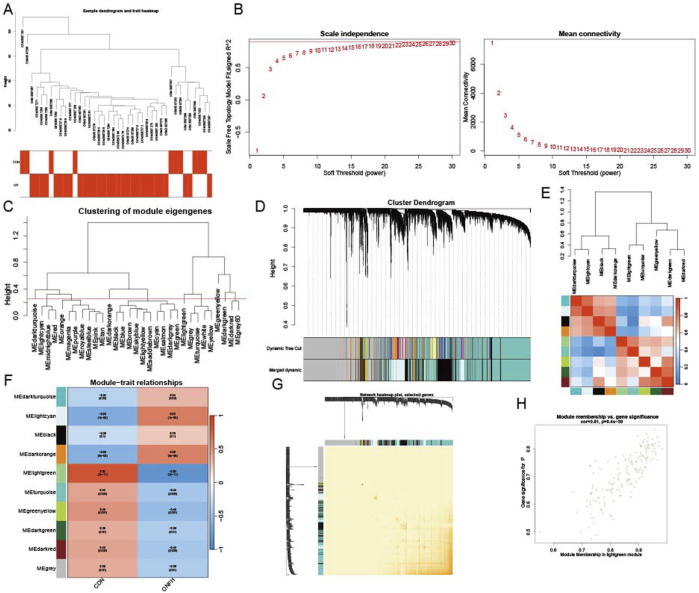
Establishing the WGCNA network. **(A)** A dendrogram of sample clustering, where individual samples are represented by tree leaves. **(B)** To get a scale-free topological fit index (*R* ^2^), a soft threshold of *β* = 19 was used (*p*-value <0.05). **(C)** To find and combine related modules, clustered dendrograms are clipped at a height of 0.25. **(D)** Modules that were original and merged are shown below the clustering tree. **(E)** Collinear heatmap of the module feature genes, where blue denotes inverse connections (correlation coefficient <−0.5) and red denotes strong correlation (correlation coefficient >0.5). **(F)** Module feature gene clustering dendrogram. **(G)** Module-trait correlations are shown in a heatmap, with blue indicating negative correlations (*p*-value <0.05) and red indicating positive correlations (*p*-value <0.05). **(H)** A scatter plot that contrasts the gene significance (GS) for controls with module membership (MM), where the statistical threshold for significance is *p*-value <0.05.

Dataset GSE123568 was obtained from the GEO database, including 10 normal and 30 SONFH samples. These samples were clustered to exclude obvious outliers using a defined threshold based on dissimilarity coefficients (Figure 4A). Outliers were identified as samples with a dissimilarity coefficient exceeding this threshold and were excluded from further analysis. Figure 4B shows a soft threshold power of *β* = 19, which was selected based on the optimal balance between achieving a scale-free topology and maintaining high network connectivity. This power value met the scale-free topology criterion with *R* ^2^ > 0.9, which is a commonly accepted threshold for network construction in WGCNA. A clustering height limit of 0.25 was used to merge strongly related modules, resulting in 31 modules identified for further analysis (Figure 4C). The consolidated modules were then displayed on the clustering tree (Figure 4D). Examination of module correlations revealed no significant associations among them (Figure 4E). To explore the relationship between modules and clinical features, correlations between module eigengene (ME) values and clinical features were analyzed. The lightgreen module exhibited a positive correlation with normal samples (*r* = 0.83, *P* = 2e−11) and a negative correlation with SONFH samples (*r* = −0.83, *P* = 2e−11) (Figure 4F). The dependability of the module delineation was validated by transcription correlation analysis inside the modules, which revealed no significant connections between the modules (Figure 4G). The lightgreen module, strongly associated with SONFH, was identified as clinically relevant, as demonstrated in the MM versus GS scatter plot (Figure 4H). Further analysis was conducted on all genes within the lightgreen module.

### Selection of feature genes and interaction analysis

**FIGURE 5:**
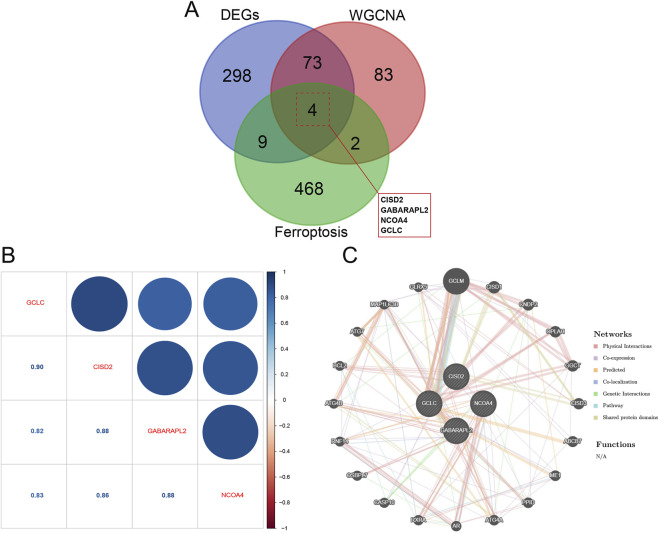
Key module genes integrated with DEGs are functionally analyzed. **(A)** Venn diagram contrasting DEGs with essential module genes. **(B)** Analysis of hub gene correlation. **(C)** Building a network of hub gene co-expression.

Four overlapping genes that are shared by all three approaches were found using a Venn diagram (Figure 5A). Gene correlations were examined, revealing both positive and negative correlations among the expressions of GCLC, GABARAPL2, CISD2, and NCOA4, as depicted in Figure 5B. This indicates significant functional relationships among these five genes. Subsequently, using the online platform GeneMANIA (http://genemania.org/), an intuitive network diagram was created to illustrate the interactions and relationships among these genes, highlighting their closely linked functions (Figure 5C).

### Modeling and testing of SONFH diagnostic nomogram

**FIGURE 6:**
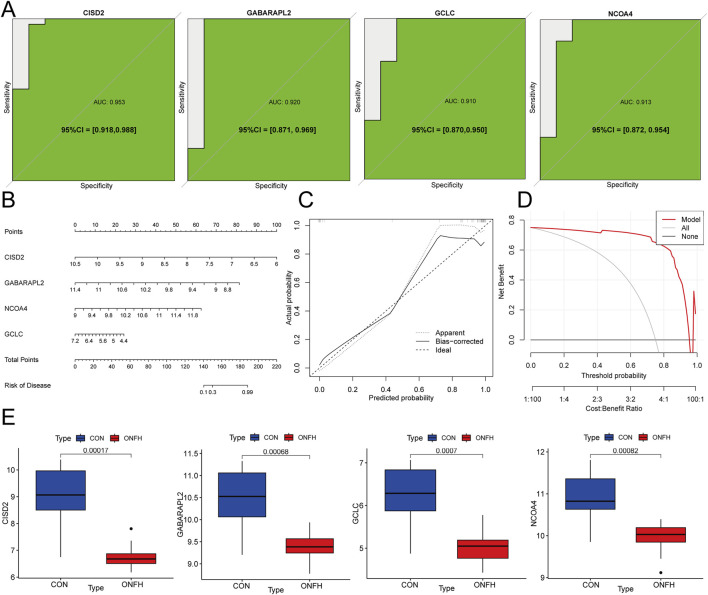
The SONFH diagnostic nomogram model was developed and validated. **(A)** ROC curve analysis of hub genes to evaluate their diagnostic performance. **(B)** Diagnostic Nomogram for predicting SONFH incidence based on hub genes. **(C)** Calibration curves to assess the degree of similarity between the predicted and true results of hub genes related to the Nomogram. **(D)** Decision Curve Analysis (DCA) curve to evaluate the clinical usefulness of the nomogram model, showing the net benefit at different threshold probabilities. DCA helps assess the model’s clinical value by comparing its performance with alternative strategies (e.g., treating all patients or no patients). **(E)** Box plot showing the expression levels of GABARAPL2, GCLC, NCOA4, and CISD2 across the samples.

To evaluate the diagnostic efficacy of GCLC, GABARAPL2, CISD2, and NCOA4, ROC analysis was performed. The obtained AUC values were as follows: GCLC (0.910), GABARAPL2 (0.920), CISD2 (0.953), and NCOA4 (0.913) (Figure 6A). These four genes were used to develop nomogram models for SONFH diagnosis using the “rms” package (Figure 6B). The predictive accuracy of the models was assessed using calibration curves, which showed minimal differences between the actual and predicted SONFH risks, indicating the high accuracy of the nomogram models (Figure 6C). The model’s accuracy was further confirmed through additional Decision Curve Analysis (Figure 6D). Validation with the GSE123568 dataset corroborated these findings (Figure 6E). These results suggest that the four key genes are involved in the pathogenesis of SONFH.

### ssGSEA analysis and correlation analysis

**FIGURE 7:**
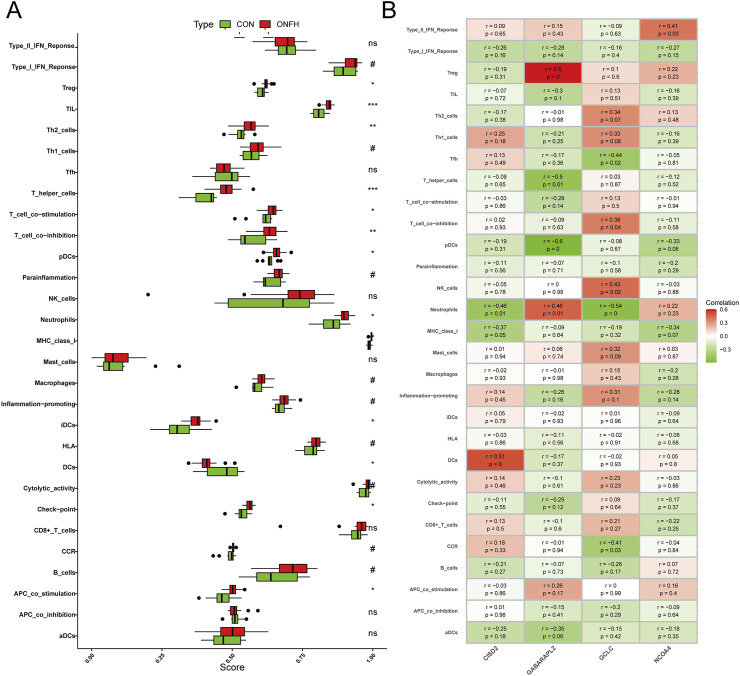
Analysis of the relationship between immune response and SONFH. **(A)** Comparison of ssGSEA scores for immune cells and pathways between the SONFH group and healthy controls. **(B)** Analysis of the correlation between immunological responses and genes specifically associated with the immune system. Statistical significance is indicated as follows: Not significant, or ns **P < 0.01 and *P < 0.001 and *P < 0.05.

Using ssGSEA, we investigated the relationship between immune infiltration in SONFH patients and healthy controls. After excluding statistically non-significant data and applying false discovery rate (FDR) correction for multiple hypothesis testing, we found that the infiltration of immune cells, such as Treg, TIL, Th2 cells, and T helper cells, was significantly higher in SONFH patients compared to the control group (Figure 7A). Next, the “corrplot” package was employed to analyze the correlation between signature genes and immune infiltration. CISD2 showed correlations with several immune functions, including DCs, neutrophils, and MHC class I. GABARAPL2 exhibited a significant positive correlation with Treg cell infiltration and a significant negative correlation with pDC cell infiltration. GCLC was associated with the infiltration of immune cells, such as Tfh, neutrophils, and NK cells. NCOA4 was solely associated with the Type II IFN response immune-related pathway (Figure 7B). These results suggest that the hallmark genes may influence immunological mechanisms throughout the progression of SONFH.

### Validation of the hub genes expression

**FIGURE 8:**
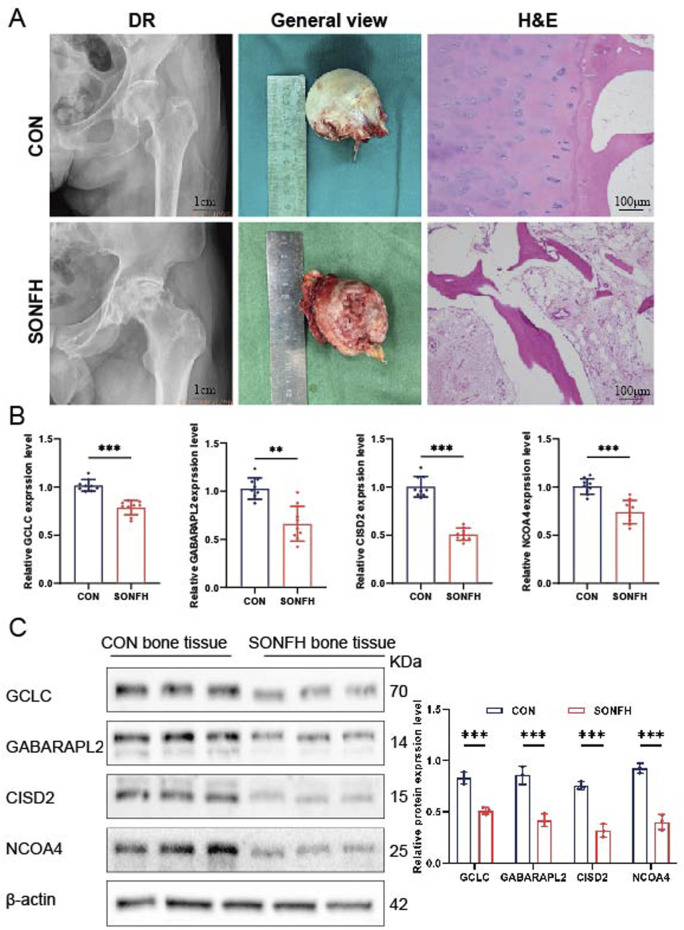
Analysis of hub gene expression in bone tissues from SONFH patients. **(A)** Representative DR scans illustrating synovial thickening. H&E staining of bone tissue in control (CON) and SONFH samples (*n* = 3). **(B)** Comparative analysis of mRNA expression levels for GCLC, GABARAPL2, CISD2, and NCOA4 in CON and SONFH samples (*n* = 9). **(C)** Quantitative assessment of protein concentrations for GCLC, GABARAPL2, CISD2, and NCOA4 in CON and SONFH samples (*n* = 3). The means ± SD are used to represent the data, while ns (not significant), *P < 0.05, **P < 0.01, and *P < 0.001 indicate significance levels.

Bone tissues were collected from nine patients diagnosed with SONFH and nine control subjects. Diagnostic radiology (DR) analysis and gross appearance revealed severe collapse of the femoral head articular surface in SONFH patients. H&E staining of SONFH bone tissue showed sparse and disordered trabeculae, with fibrous tissue hyperplasia, myxoid degeneration, necrosis, and hemorrhage observed between the trabeculae (Figure 8A). qRT-PCR analysis revealed decreased expression of GCLC, GABARAPL2, NCOA4, and CISD2 in the SONFH patients (Figure 8B, *n* = 9). Western blot analysis further confirmed the downregulation of GCLC, GABARAPL2, NCOA4, and CISD2 in the SONFH group compared to the control group (Figure 8C, *n* = 3). The relatively small sample size (*n* = 3) for Western blot analysis is a limitation and may affect the generalizability of the quantitative findings. These findings collectively demonstrate significant differences in the expression levels of GCLC, GABARAPL2, NCOA4, and CISD2 between SONFH and control bone tissue.

### Identification of candidate drugs

A comprehensive understanding of the structural characteristics influencing receptor sensitivity requires evaluating protein–drug interactions (Al-Mustanjid et al., 2020; Mahmud et al., 2020). Using transcriptome data from the DSigDB database, we utilized Enrichr to identify 86 candidate pharmacological compounds associated with hub genes, which could serve as potential therapeutic targets for SONFH. The top 10 compounds were selected based on their corrected *P*-values, and they hold promise as potential treatments for SONFH, targeting the identified hub genes. The following compounds were identified: Diethyl sulfate: Known to inhibit specific cellular processes, Diethyl sulfate demonstrates anti-inflammatory properties that may help reduce the inflammatory component of SONFH. Meloxicam: A nonsteroidal anti-inflammatory drug (NSAID), Meloxicam targets cyclooxygenase enzymes (COX-1 and COX-2) to alleviate inflammation and pain, making it a viable therapeutic option for managing SONFH-related inflammation. IMD-0354: A potent NF-kB inhibitor, IMD-0354 interferes with the inflammatory and immune response pathways, showing therapeutic potential in SONFH. These pharmacological agents, specific to the hub genes identified in the DSigDB database, are summarized in Table 2. The compounds listed were predicted *in silico* based on known drug-gene interactions and signature analyses from the DSigDB database.

## Discussion

SONFH is a progressive and refractory orthopedic disease that significantly impacts patients’ quality of life. Although pathological changes such as bone trabeculae damage and inadequate cell replacement are observed early in the disease, clinical symptoms are often subtle. As the disease advances, femoral head sclerosis, cystic changes, and potential collapse lead to substantial impairments (Zhao et al., 2015; Petek et al., 2019). Chen et al. (2020) demonstrated that steroid-induced SONFH in rats is associated with increased ROS levels and osteoclast alterations, both of which contribute to the disease’s pathophysiology. Previous studies have established that ROS, iron accumulation, lipid peroxidation, and glutathione depletion are crucial in ferroptosis (Hirschhorn and Stockwell, 2019). Furthermore, recent findings by Sun et al. (2022) showed that dexamethasone induces ferroptosis in glucocorticoid-induced osteonecrosis of the femoral head via the P53/SLC7A11/GPX4 pathway. However, studies exploring ferroptosis-related biomarkers and pathways in SONFH progression remain limited. Our research investigates novel genes involved in ferroptosis and SONFH, offering valuable insights for clinical diagnosis and potential therapeutic strategies.

In this study, we identified key ferroptosis-related genes through bioinformatics analysis of the GSE123568 dataset. Using WGCNA, we identified lightgreen module genes most associated with SONFH. Since these genes underwent functional enrichment, additional pathway analysis was not repeated to avoid redundancy. By intersecting these genes with ferroptosis-related genes from the FerrDb database, we identified four key hub genes—GCLC, GABARAPL2, NCOA4, and CISD2—that improve our understanding of steroid-induced SONFH mechanisms. These biomarkers showed strong diagnostic potential for SONFH in the nomogram, with clinical validation confirming significant expression differences between SONFH and control bone tissues. Immune infiltration analysis revealed strong correlations between these biomarkers and immune cell functions. Additionally, we identified potential drugs targeting these genes, such as Diethyl sulfate, Meloxicam, and NIMUSTINE, which may aid in drug development for SONFH. However, drug repositioning has limitations. These agents show promise but may have off-target effects. Diethyl sulfate, known for its alkylating properties, could affect other cellular pathways. NIMUSTINE, a chemotherapy drug, is associated with significant toxicity, limiting its use in non-cancer conditions like SONFH. Further clinical trials are needed to assess their safety and efficacy in SONFH patients.

The glutamate-cysteine ligase catalytic subunit (GCLC), a key part of glutamate-cysteine ligase, helps prevent ferroptosis by maintaining glutamate balance during cystine shortage (Zuo et al., 2023). GCLC is also linked to the infiltration of various immune cell types, as well as levels of immunostimulatory and immunosuppressive agents and chemokines. Increased GCLC expression boosts glutathione production, reduces CD36 overexpression, lessens ferroptosis in cytotoxic T lymphocytes (CTLs), and enhances their antitumor activity (Han et al., 2024). A recent study showed that GCLC overexpression has antioxidant and anti-apoptotic effects (Zhang et al., 2022), which aligns with findings showing that increased GCLC expression via 1, 25(OH)2D3 also prevents apoptosis (Kanikarla-Marie and Jain, 2016). Additionally, GCLC and glutathione (GSH) are crucial for osteoclast differentiation and bone resorption in mice (Hu et al., 2025). However, we observed that GCLC expression was reduced in SONFH bone tissue, suggesting that GCLC may regulate the ferroptosis process and play a role in the development of SONFH.

In mammals, selective autophagy cargos are recognized by ATG8 proteins, which include two subfamilies: LC3 (LC3A, LC3B, and LC3C) and GABARAP (GABARAP, GABARAPL1, and GABARAPL2) (Yamamoto et al., 2021). GABARAPL2, part of the GABARAP family, is involved in autophagosome maturation and degradation (Hervouet et al., 2015). In mitochondrial autophagy, GABARAPL2 helps remove damaged mitochondria, maintaining cellular energy balance and preventing excessive ROS production (Antón et al., 2016). High mRNA expression of GABARAPL2 has been linked to better overall survival in renal cancer but worse overall survival in head and neck cancer (Uhlen et al., 2017). In mice, the loss of GABARAPL2 leads to excessive activation of caspase-11 inflammasomes, causing a destructive immune response (Eren et al., 2020; Sakaguchi et al., 2020). Our study found that GABARAPL2 expression is reduced in SONFH bone tissue. Additionally, ROC curve analysis showed that GABARAPL2 has strong diagnostic potential for SONFH (AUC = 0.920). Based on these results, we suggest that GABARAPL2 may be a useful biomarker for diagnosing SONFH.

Human health and disease are influenced by the 3Cys-1His [2Fe–2S]-binding CDGSH domain in the iron-sulfur proteins of the NEET protein family (Wiley et al., 2007). CISD2, also known as NAF-1, is encoded by a gene located in the endoplasmic reticulum and outer mitochondrial membrane. In mammals, CISD2 regulates autophagy, oxidative stress, calcium and iron balance, and longevity (Shao et al., 2020; Wang et al., 2014). As a well-studied member of the NEET family, CISD2 has been linked to diseases like diabetes, obesity, aging, and neurodegeneration, and is increasingly recognized as a potential cancer treatment target (Kusminski et al., 2012; Chen et al., 2009). In our study, we found that CISD2 showed strong diagnostic value for SONFH (AUC = 0.953) and was significantly downregulated in SONFH bone tissues. Based on these results, we propose CISD2 as a promising biomarker for diagnosing SONFH.

Nuclear receptor coactivator 4 (NCOA4) is a cargo receptor for ferritin, selectively transporting it to autophagosomes in a process called ferritinophagy, which has attracted significant research interest (Mancias et al., 2014). NCOA4 also regulates the intracellular labile iron pool (LIP) and plays a key role in iron-related processes, including homeostasis, transport, metabolism, release, and utilization (Le et al., 2024). Recent studies have shown that NCOA4 is involved in several important physiological and pathological processes, affecting the development of various disorders (Dai et al., 2024; Dai et al., 2023; Federico et al., 2022; Santana-Codina et al., 2022; Jin et al., 2023). NCOA4 levels can be influenced by certain conditions. For example, a high-fat diet (HFD) in mice causes abnormal liver fat accumulation, reduces iron levels, and increases endoplasmic reticulum stress by accumulating p62, which disrupts NCOA4 and ferritin expression (Jiang et al., 2020). Research is ongoing on how hypoxia affects NCOA4, with one study indicating that hypoxia promotes ferroptosis by increasing mitochondrial ferritin and decreasing NCOA4 levels in macrophages (Fuhrmann et al., 2020). We also found that NCOA4 showed strong diagnostic value for SONFH (AUC = 0.913) but was weakly expressed in the bone tissues of SONFH patients. Based on these results, we propose NCOA4 as a potential biomarker for SONFH diagnosis.

### Limitations

This study, despite the strong diagnostic predictive ability of our nomogram model based on four genes for SONFH patients, has several limitations. First, several key clinical factors such as sex, age, and comorbidities were not included because the data used was derived from a database that lacks detailed clinical information. This highlights the need for future clinical studies to incorporate these variables to improve the model’s generalizability. Second, the biological functions of the identified genes and their roles in SONFH remain unclear. While we identified genes strongly linked to SONFH, their exact mechanistic contributions require further investigation. Third, the retrospective nature of the GEO data and the potential for sampling bias limit the conclusions that can be drawn. The dataset used was not randomized and might have inherent biases due to patient selection, which may affect the generalizability of our findings. Finally, the clinical validation of the model was constrained by a small sample size, which may introduce bias. Additionally, the lack of *in vivo* validation further limits the confidence in the applicability of the model in a clinical setting. To address these limitations, we plan to expand patient recruitment in future studies and incorporate *in vivo* models to strengthen the model’s reliability and applicability.

## Conclusion

In summary, GCLC, GABARAPL2, NCOA4, and CISD2 could serve as potential biomarkers for SONFH patients. These biomarkers may aid in early diagnosis, risk assessment, and tracking disease progression. They also show promise as drug targets, offering opportunities for new therapeutic strategies. However, clinical use of these biomarkers requires validation in larger, prospective studies and *in vivo* models. Future research should address limitations such as small sample size and retrospective data, and include longitudinal studies to better evaluate their clinical value.

## Data Availability

The datasets presented in this study can be found in online repositories. The names of the repository/repositories and accession number(s) can be found in the article/Supplementary Material.
